# Development of an Optical Sensor Using a Molecularly Imprinted Polymer as a Selective Extracting Agent for the Direct Quantification of Tartrazine in Real Water Samples

**DOI:** 10.3390/polym16060733

**Published:** 2024-03-07

**Authors:** Gerson A. Ruiz-Córdova, Jaime Vega-Chacón, Maria del Pilar Taboada Sotomayor, Juan C. Tuesta, Sabir Khan, Gino Picasso

**Affiliations:** 1 Technology of Materials for Environmental Remediation (TecMARA) Research Group, Faculty of Sciences, National University of Engineering, Lima 15333, Peru; gerson.ruiz.c@uni.pe (G.A.R.-C.); jvegac@uni.edu.pe (J.V.-C.); 2 Institute of Chemistry, State University of São Paulo (UNESP), Araraquara 01049-010, SP, Brazil; m.sotomayor@unesp.br; 3 National Institute of Alternative Technologies for Detection, Toxicological Evaluation and Removal of Micropollutants and Radioactives (INCT-DATREM), Araraquara 14800-900, SP, Brazil; 4 Laboratorio de Biotecnología, Universidad Nacional Autónoma de Alto Amazonas, Calle Prolongación Libertad 1220, Yurimaguas 16501, Peru; jtuesta@unaaa.edu.pe; 5 Department of Natural Sciences, Mathematics, and Statistics, Federal Rural University of the Semi-Arid Region, Mossoro 59625-900, RN, Brazil; sabir@ufersa.edu.br; 6 Department of Exact Sciences and Technology, State University of Santa Cruz, Ilhéus 45662-900, BA, Brazil

**Keywords:** functionalized fiber, MIP, tartrazine, precipitation method

## Abstract

This study presents a new optical sensor for tartrazine (TAR) quantification developed using a molecularly imprinted polymer (MIP) as the recognition element, with optical fiber serving as the supporting substrate. The fiber surface was functionalized with 3-(trimethoxysilyl)propyl methacrylate (MPS), and the fiber was coated with MIP using the precipitation polymerization method. The analysis of MIP immobilization on the functionalized optical fiber (FF) was conducted through the use of scanning electron microscopy (SEM) and Fourier transform infrared spectroscopy (FTIR) techniques. Experimental parameters, such as contact time and fiber length, were adjusted in order to obtain the highest sensitive response signal for the functionalized optical fiber (FF-MIP). The fiber sensor, FF-MIP, exhibited a relatively higher response signal for tartrazine compared to other interfering dyes. The rapid and total desorption of the analyte from FF-MIP allowed the immediate reemployment of FF-MIP, which also presented an acceptable repeatability for the reflectance signal. The imprinting factors for the studied dyes were between 0.112 and 0.936 in front of TAR, 1.405, and selectivity factors were between 1.501 and 12.545, confirming the sensor selectivity. The FF-MIP sensor was successfully applied for tartrazine quantification in real water samples, where it yielded satisfactory results comparable to those of the HPLC reference method.

## 1. Introduction

Tartrazine, also referred to as E102 or acid yellow 23, is a highly popular dye that is widely employed as a color additive in the food industry; when applied, the dye confers a lemon/yellow hue to food products, including jellies, condiments, cereals, snack foods and beverages [1]. Classified as an azo compound with aromatic rings, the widespread use of tartrazine raises serious concerns due to its potential danger to humans. Exposure to tartrazine has been associated with allergic reactions, particularly urticaria, especially in individuals with asthma. Apart from allergic reactions, studies reported in the literature have also linked tartrazine to Attention Deficit Hyperactivity Disorder (ADHD) in children, hives and insomnia, and in certain cases, the compound has been associated with an elevated risk of developing cancerous tumors [2,3]. Thus, careful monitoring and determination of tartrazine in food products is essential for protecting and safeguarding public health. Conventional methods generally employed for tartrazine quantification in food products include high-performance liquid chromatography (HPLC) [4,5,6], electrophoresis [7,8], and spectrophotometry [9]. While these methods offer the required sensitivity and selectivity for the effective analysis of samples with complex matrices, they are often time-consuming and require the use of organic solvents and substantial human resources. In this sense, developing rapid, straightforward and simple alternative methods for the quantitative determination of dyes in water bodies that are less costly yet highly efficient still remains a crucial objective among researchers in the field. In this context, the use of a sensing platform developed using a molecularly imprinted polymer (MIP) supported on the surface of an optical fiber, combined with a swift and cost-effective quantitative technique like UV-Vis spectroscopy, emerges as an interesting alternative technique [10].

MIPs are materials constructed around a molecule of interest (analyte/template), where the analyte is subsequently removed in order to generate a cavity, conferring high selectivity to the polymer, properties that can be used with various forms of materials and extensive applications [11,12,13]. The imprinting process involves the interaction of the template molecule with a chosen functional monomer, leading to the formation of complexes. Afterward, a cross-linking agent (structural monomer) and an initiator are introduced, stimulating the beginning of polymerization and the formation of a three-dimensional network [14,15]. The template molecules are then extracted, leaving behind cavities with dimensions and functional groups complementary to the templates, where selective interactions are ensured between the template molecules and the polymer, often via hydrogen bonds or dipole–dipole interactions.

Earlier advancements in the use of MIPs for quantitative applications have involved the adaptation of MIPs as recognition elements for various detection techniques [16,17,18,19,20]. The implementation mechanism includes immobilizing the MIP on different supporting materials (e.g., optical fibers, carbon-based electrodes, etc.) [21,22], which helps increase the surface area in contact with the solution and improves the access of the analyte to the selective cavities. Depending on the nature of the supporting material, its specific properties (e.g., optical or electrochemical properties) can be leveraged by using suitable detection techniques, such as UV-visible, Raman and Fluorescence Spectroscopy, for the successful detection of the analytes of interest [17,18,19]. With the dyes providing an intense color in the visible spectrum and the fiber endowed with optical properties, using visible spectroscopy, one can expect to produce highly sensitive, efficient and cost-effective analyses targeted at the determination of complex matrices.

In previous work [23], SiO_2_@MIP material was studied for the quantification of tartrazine with high selectivity. Based on these findings, a novel optical sensor formed by an optical fiber covered with a molecularly imprinted polymer (FF-MIP) was developed for rapid, selective and highly efficient quantification of tartrazine in water matrices. Synthesis optimization analysis was performed in order to obtain a sensor with the highest signal. The proposed FF-MIP material exhibited excellent performance in terms of accuracy and precision, with the additional advantages of simplicity, rapidity and cost-effectiveness. The findings of this study show that the FF-MIP sensor can be readily applied for tartrazine monitoring in water samples with a view to preventing the occurrence of allergic reactions in some susceptible patients and helping to control the adverse effects of TAR when employed as a color additive in food products.

## 2. Experimental Section

### 2.1. Chemicals and Solutions

The starting materials employed in the experiments included the following: tartrazine (TAR) sodium salt used as an analyte, *N-N′*-methylene-bis-acrylamide (MBAA) used as a structural monomer, potassium persulfate (KPS) used as a radical initiator and 3-(trimethoxysilyl)propylmethacrylate (MPS) as a functional agent for the optical fiber; all these materials, which were acquired from Sigma-Aldrich (St. Louis, MO, USA), were of analytical quality. The interferents employed in the selectivity studies were as follows: toluene, Sunset Yellow (SY), Curcumin (C), Rhodamine B (RB), Toluidine Blue (TB), Methylene Blue (MB) and Acid Blue 29 (AB); all of these materials, which were purchased from Sigma-Aldrich^®^, were of analytical quality and used as received. Acrylamide (AA), which was employed as a functional monomer, and ammonia, hydrochloric acid (HCl) and sodium hydroxide (NaOH) were purchased from Merck^®^ (Lima, Peru). Deionized water was obtained from the Milli-Q-Direct-0.3 equipment (Millipore, provided by Merck) with a conductivity of 8 μS cm^−1^. Silica-based optical fibers of 1 mm of diameter were acquired from Thorlabs^®^.

### 2.2. Instrumentation

UV-Vis spectra were acquired using a Cary 60 UV-Vis spectrophotometer (Agilent Technologies, Santa Clara, CA, USA) connected to a set of Agilent optical fibers purchased with the instrument, facilitated by an adapter crafted in our laboratory for this function particular purpose. Fourier transform infrared (FTIR) spectra were obtained using an ALPHA II spectrophotometer (Bruker, Billerica, MA, USA). The samples were dried at room temperature prior to the analysis. The spectra were scanned over the range of 4000 to 400 cm^−1^ in the ATR mode. Scanning electron microscopy (SEM) analyses were performed using a JSM-7500F microscope (JEOL, Tokyo, Japan). Thermogravimetric analysis (TGA) was carried out using the STA 6000 simultaneous thermal analyzer (Perkin Elmer, Waltham, MA, USA). The temperature program was set up under the following conditions: temperature level from 35 °C to 900 °C; heating rate of 9 °C min^−1^; under a nitrogen atmosphere. The derivative thermogravimetry curve (DTG) was obtained from the TGA curve. Optical images of the fibers were obtained using Stemi 508 doc stereomicroscope (Carl Zeiss, San Diego, CA, USA). For comparative purposes, the UV-Vis/HPLC quantification technique was used as the reference method; the technique was performed using a Thermo Fisher Scientific Vanquish chromatograph (Waltham, MA, USA). In the UV-Vis/HPLC analysis, specific conditions were applied; these included the use of a C18 column (250 × 4.6 mm, 5 µm particle size, and 100 Å pore size-Kinetex). The mobile phase consisted of a mixture of methanol:water (20:80 *v*/*v*), with a flow rate of 1 mL min^−1^. A volume of 20 µL from the sample or standard was injected, and the signal was recorded at a wavelength of 430 nm.

### 2.3. Functionalization of Optical Fiber (FF)

The optical fiber was functionalized in order to incorporate methacrylate groups into the material; this was carried out to promote polymerization on the surface [14]. The optical fiber was washed with 5% HNO_3_ solution, dried at room temperature and submerged in a solution containing 500 µL of MPS and 1000 µL of toluene. The material was subsequently sealed and stirred for 12 h. After that, the fiber was removed and subjected to washing with ethanol and water and left to air-dry at room temperature. At the end of this procedure, the functionalized fiber was ready to be used for the polymerization of the MIP (Figure S1).

### 2.4. Synthesis of Molecularly Imprinted Polymer (MIP) on the Surface of the Functionalized Optical Fiber (FF-MIP)

**Figure 1:**
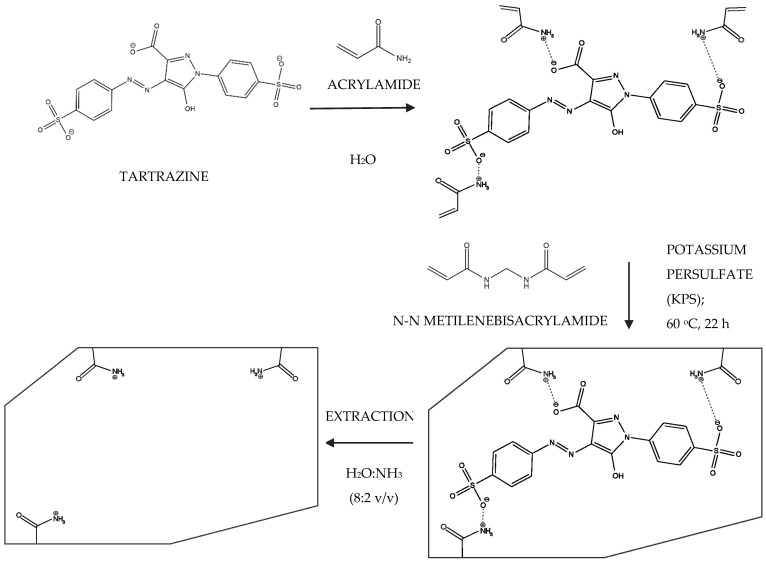
Scheme illustrating the synthesis of the FF-MIP and FF-NIP materials for tartrazine determination.

The MIP was synthesized using the precipitation method, which was adapted from the work of Ruiz et al. [23]. To perform the synthesis, 0.1 mmol of TAR (template molecule) and 0.2 mmol of AA (functional monomer) were blended in 4 mL of deionized water. The functionalized optical fiber (FF) was added to the solution, and the mixture was allowed to rest for 2 h in order to facilitate the interaction between TAR and AA. Thereafter, 10 mmol of MBAA was placed into the system. The system was then purged with nitrogen for 15 min, and 0.185 mmol of KPS was added therein. The system was subsequently sealed in an anaerobic atmosphere and placed in a water bath at 60 °C for 3 h. After this period, the fiber was washed using a 20% ammonia solution in order to remove the entrapped TAR from the polymeric network on the surface of the fiber. Finally, the fiber coated with the MIP (FF-MIP) was washed with deionized water and left to dry at room temperature for 24 h. In parallel, for comparison purposes, a fiber coated with a NIP was prepared using the same procedure employed for the synthesis of the FF-MIP but without the presence of the analyte; this material was named FF-NIP. Figure 1 presents a brief scheme illustrating the preparation of the fibers coated with the MIP and NIP.

### 2.5. Optimization of Reflectance Response of FF-MIP Sensor

**Figure 2:**
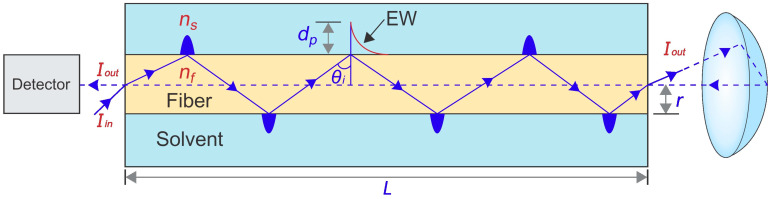
Scheme of attenuated total internal reflection of the light beam into the optical fiber and the solvent (acetonitrile).

In attenuated total internal reflection spectroscopy (ATR), we used optical fibers as a base and measured reflectance as the response signal. This technique is important for understanding how the signals are acquired. Essentially, when a light beam propagates from a material with a high refractive index, such as an optical fiber, to another material with a low refractive index, such as acetonitrile, at an angle *θ_i_*, internal reflection of the light beam occurs. The reflected light returns to the high refractive index material, generating an evanescent wave (EW) in the low refractive index material, which decays exponentially. This wave exists in the proximity of the interface between the two materials, extending approximately one-third of the wavelength of the incident light in the material with the low refractive index (Figure 2) [24,25,26].

When using the fiber without any coating, attenuation of the evanescent wave occurs at the fiber–analyte interface due to the interaction between the analyte and the EW. This interaction leads to variations in the detected light intensity, which correlates with the optical properties of the adsorbent medium (acetonitrile). The transmission of light through the solvent can be described by the Lambert–Beer Law:

(1) $$I_{out}=I_{in}e^{-\xi \left(n_{s}\right)L}$$

(2) $$\xi \left(n_{s}\right)=\frac{n_{s}\tau \lambda }{2r\pi \left(n_{f}^{2}-n_{s}^{2}\right)}\cos\theta_{i}\cot\theta_{i}$$

Here, *I_in_* represents the incident light intensity, *I_out_* denotes the output light intensity that returns to the equipment detector for corresponding measurements, *L* is the length of the fiber, *ξ_(ns)_* stands for the extinction coefficient of the evanescent wave in the solvent, *n_s_* indicates the refractive index of the solvent, *τ* is the extension coefficient of the analyte, *λ* is the wavelength of the light directed towards the fibers, *r* symbolizes the fiber radius, *n_f_* is the refractive index of the fiber, and *θ_i_* represents the angle of incidence of the light at the fiber–solvent interface [24].

If the angle of incidence *θ_i_* exceeds the critical angle, total internal reflection occurs, and the penetration depth, *d_p_*, of the generated EW can be quantified using the following equation [25,26]:

(3) $$d_{p}=\frac{\lambda }{2\pi \;\sqrt{n_{f}^{2}\;sin^{2}\theta_{i}-n_{s}^{2}}}$$

For the adsorption analysis in this work, the solvent was acetonitrile, and its refractive index (*n_s_*) remained constant throughout the adsorption analysis, as no TAR was introduced into the solvent, and TAR is inherently insoluble in acetonitrile. The parameters under investigation included *n_f_*, representing the refractive index of the fiber, which correlates directly with the quantity of TAR adsorbed (TAR concentration) onto the fiber, and *L*, denoting the length of the fiber immersed in the TAR solution.

In order to determine the optimal parameters for variation in the reflectance signal of the FF-MIP and FF-NIP fibers, a solution of 1.0 mmol L^−1^ tartrazine was prepared in the presence of 0.01 mol L^−1^ phosphate solution at pH = 3. A 5 mL volume flask was filled up with the aforementioned solution, and the FF-MIP was placed therein; the mixture was then sealed and magnetically stirred for a certain contact time. Afterward, the FF-MIP was removed, and the excess tartrazine solution was cleaned with acetonitrile. Finally, the fiber was inserted into an optical fiber module connected to a UV-Vis spectrophotometer, and reflectance measurements were conducted at 430 nm in acetonitrile. In this work, the reflectance variation (Δ*R%*) was evaluated and calculated by the difference between the sample reflectance and the blank reflectance (Equation (4)).

(4) $$\Delta R{\%}_{i}=R{\%}_{i}-R{\%}_{BK}$$

Based on this procedure, the contact time was evaluated in triplicate in the range of 1 to 20 min and at 3 different TAR concentrations (1.0, 2.0 and 4.0 mmol L^−1^). After obtaining the optimal contact time, the influence of the immersion length of the fiber was studied. The applied experimental conditions were TAR concentrations ranging from 0.25 and 20 mmol L^−1^ and varying immersed lengths of 28 mm (completely immersed), 22, 18 and 12 mm for both fibers, FF-MIP and FF-NIP.

### 2.6. Reusability and Repeatability of FF-MIP and FF-NIP Sensors

The use of optical fibers as sensing platforms enhances the mass transfer capability of the material. Taking advantage of this characteristic, desorption tests were carried out by eluting adsorbed TAR from the fibers in 1 mL of 0.01 mol L^−1^ phosphate solution for different time periods (3–15 s). The absorbance of the obtained solution was measured at a wavelength of 430 nm, and the maximum value was taken as 100% relative desorption for comparison purposes.

For repeatability analysis, FF-MIP and FF-NIP were evaluated by measuring reflectance at 430 nm using solutions of 1, 2.5, and 7.5 mmol L^−1^ of tartrazine at pH 3. No calculations for FF-NIP were performed at 7.5 mmol L^−1^ concentration because this concentration fell outside the working range for this fiber. The obtained data were then used to compare the relative standard deviations (RSD) with Horwitz RSD (*RSD_Horwitz_*–Equation (5)) [27,28] for each fiber and concentration, and the repeatability was confirmed if *RSD_exp_* < *RSD_Horwitz_.*

(5) $$RSD_{Horwitz}\left(\%\right)=0.67\times 2^{1-0.5\times \log\left[TAR\right]}$$

### 2.7. Calibration Curve

The calibration curve was plotted under optimized conditions. In order to plot the calibration curve, different TAR solutions were evaluated in the range of 0.25 to 10 mmol L^−1^ using acetonitrile as a solvent for TAR quantification. The limit of detection (LOD) and limit of quantification (LOQ) were determined using the first point of the calibration curve, as reported in previous studies [29,30,31].

### 2.8. Selectivity Tests

To perform the selectivity analyses, some dyes were selected; the dyes chosen for the analyses included the following: Sunset Yellow (SY), Curcumin (C), Rhodamine B (RB), Toluidine Blue (TB), Methylene Blue (MB), and Acid Blue 29 (AB). The solutions were prepared based on the same procedure using 1 mmol L^−1^ concentration for each dye and phosphate solution at pH 3. All the reflectance variations measurements (Δ*R%*) were performed at 430 nm and in triplicate in order to evaluate the selectivity of the materials when it comes to the analyte detection; for comparison purposes, 100% of relative retention was considered the maximum level of TAR reflectance variation, and the corresponding values for the selected dyes were calculated proportionally. The molecular imprinting factor (*α*) and the selectivity factor (*β*) [23] were calculated using the following equations:

(6) $$\alpha =\frac{\Delta R{\%}_{FF-MIP}}{\Delta R{\%}_{FF-NIP}}$$

(7) $$\beta =\frac{\alpha_{TAR}}{\alpha_{Interferent}}$$

The molecular imprinting factor (*α*) reflects the ability of the cavity formed in MIP synthesis to recognize the molecule of interest relative to the NIP (Equation (6)); hence, values above 1 are expected to indicate a good selective material. The selectivity factor (*β*) is the relation between the imprinting factor of the analyte and that of the interfering compounds (Equation (7)); *β* values above 1 are deemed suitable since the imprinting factor of the analyte is required to be higher than that of the other compounds in a highly selective material.

### 2.9. Application of the FF-MIP

To evaluate the efficiency of the FF-MIP, the material was employed for TAR quantification in real water samples. The analysis was conducted using the following samples: two real water samples from piped water and bottled water for human consumption; two samples of river water from the Millpu River and Cachi River (Ayacucho, Peru), and one sample of industrial residual water obtained from the city of Ayacucho, Peru; all the water samples were spiked with a defined concentration of tartrazine and were tested using the proposed UV-Vis reflectance spectrophotometry method and the HLPC method (reference method) for comparison purposes.

## 3. Results and Discussion

### 3.1. Morphological and FTIR Characterization of the FF-MIP and FF-NIP

**Figure 3:**
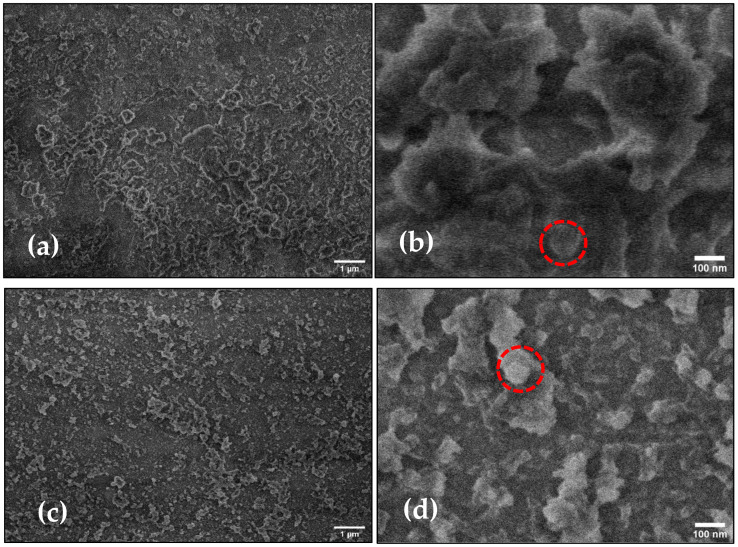
SEM micrographs of the FF-NIP and FF-MIP materials in different magnifications: (**a**) ×10,000, (**b**) ×50,000 for the FF-NIP, (**c**) ×10,000, and (**d**) ×50,000 for the FF-MIP.

Figure 3 shows the SEM micrographs obtained for the FF-MIP and the FF-NIP. Even though the FF-MIP and FF-NIP materials appeared to have relatively similar particle sizes (red circles in Figure 3), the FF-NIP material exhibited more agglomerated particles compared to the FF-MIP; this is attributed to the irregular (non-orderly) growth of polymer on the FF-NIP due to the absence of the analyte, which is an important structural element for polymerization. This irregular polymer growth effect provoked a small degree of roughness in the FF-NIP material, as shown in Figure 3; similar images have been reported for other types of MIP materials [32,33]. The SEM images point to the efficient polymerization and immobilization of the MIP on the surface of optical fiber.

**Figure 4:**
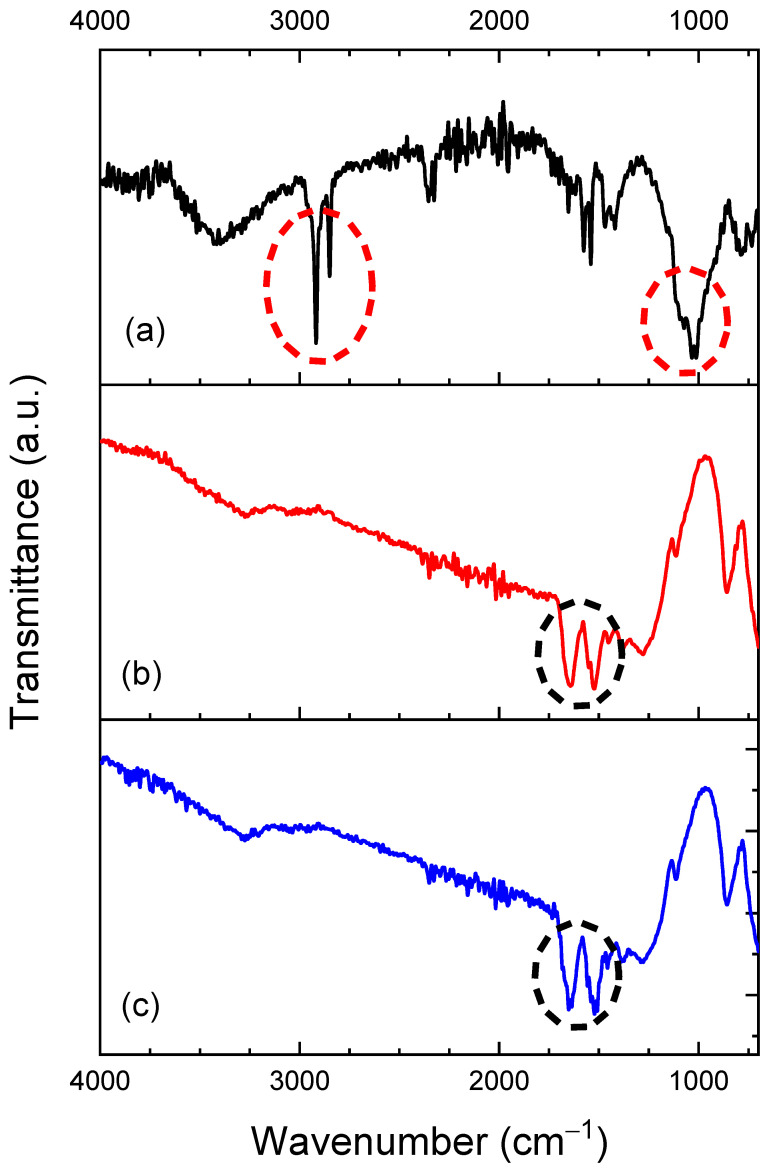
FTIR spectra of (**a**) FF, (**b**) FF−MIP, and (**c**) FF−NIP.

The FTIR spectra of the FF, FF-MIP and FF-NIP are depicted in Figure 4. Looking at the FF spectra, the presence of a characteristic band at 1030 cm^−1^ with a shoulder at 1115 cm^−1^, both corresponding to the stretching asymmetric Si-O-Si vibrations of silica [33,34]. In all three samples, the band situated at 1650 cm^−1^ corresponds to C=C from MPS, while the bands at 2950, 1475, 1415, and 780 cm^−1^ correspond to C-H stretching vibrations from CH_2_ and CH_3_ present in MPS [35,36,37]. On the other hand, for the FF-MIP and FF-NIP spectra, the bands located at 3500 and 1654 cm^−1^ are possibly related to the N-H and C-O groups from MBAA (from the polymeric structure), while the band at 1516 cm^−1^ corresponds to the N-H secondary amide group from the MBAA polymer [10,33,38,39,40,41]. These bands were also observed in the FTIR spectra of the bulk MIP and NIP materials (Figure S2). The absence of sp^3^ C-H bond at 2950 cm^−1^ and the band corresponding to Si-O-Si vibrations of silica in the FF-MIP and FF-NIP points to the successful immobilization of the polymers on the fiber surface [41]. It is worth noting that, prior to the analysis, FF-MIP was washed in order to remove the analyte, so the FTIR profiles of FF-NIP and FF-MIP were expected to exhibit some similarities.

As can be observed in Figure S3, the different thermogravimetric profile results obtained for the MIP and NIP bulk are practically the same. Based on this analysis, the compositions of the MIP and NIP were found to be identical, and the only difference between them lay in the presence of cavities formed in the MIP, which were undetectable in the thermogravimetric analyses. The first stage of thermal decomposition was attributed to the volatilization of water and ammonia used in the removal of the analyte; the percentage weight losses recorded for the MIP and NIP were c.a. 10% and 20%, respectively, for the temperature range of 35 °C to 140 °C [42]. The second stage of thermal decomposition resulted in 75% and 65% mass loss for the MIP and NIP, respectively, based on the DTG curves; this may be associated with a decomposition in two steps for both AA and MBAA: the first weight loss was associated with the aliphatic alkene groups [43,44] and the second weight loss was associated with the carboxyl groups and the amine groups [43,44,45]. MBAA was responsible for the greatest mass loss since it is present in a much higher proportion in the polymerization process than AA (ratio of AA:MBAA = 2/100). This analysis ensures the thermal stability of the polymers immobilized on the FF-MIP and FF-NIP fibers.

### 3.2. Optimization of the Sensor Response

**Figure 5:**
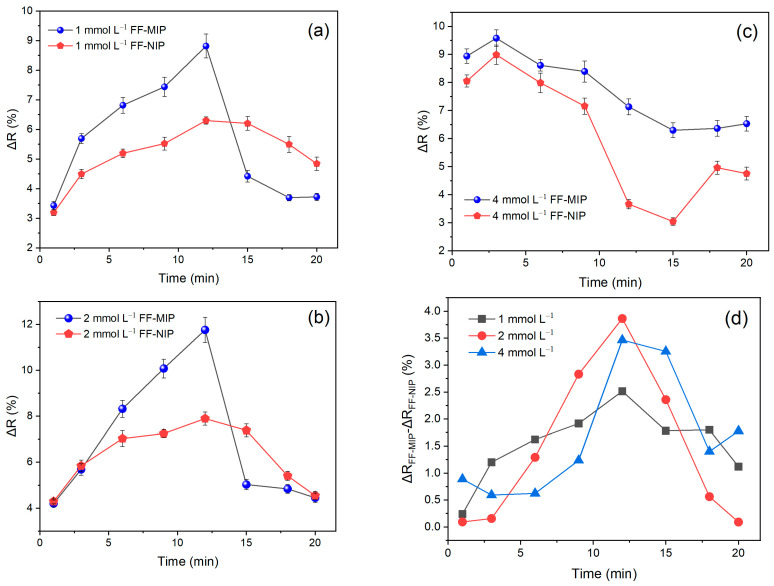
Reflectance variation profiles of FF−MIP and FF−NIP at different adsorption times for TAR concentrations of (**a**) 1.0 mmol L^−1^, (**b**) 2.0 mmol L^−1^, and (**c**) 4.0 mmol L^−1^ (n = 3). (**d**) Difference between reflectance variation of FF−MIP and FF−NIP at different contact times and TAR concentrations.

The reflectance variation (Δ*R%*) reached its maximum at 12 min before sharply declining; this was observed at concentrations of 1.0 and 2.0 mmol L^−1^ of TAR (Figure 5a,b). However, at 4.0 mmol L^−1^ (Figure 5c), the signal began to decrease at 3 min. This phenomenon arises due to the light propagation through the fiber via ATR, which generates evanescent waves. According to Equation (3), the increase in the refractive index of the fiber results in a diminution in the penetration depth of EW, as does the extinction coefficient of the evanescent wave, *ξ(n_s_)* (Equation (2)). This, in turn, reduces the interaction between the light beam and the analyte. As a consequence, the reflectance variation diminishes. Moreover, this increase in the refractive index is correlated with the saturation of the analyte on the fiber, which is dependent on the adsorption time (contact time) and the TAR concentration analyzed.

To determinate the optimal contact time, the difference in reflectance variation (Δ*R%*) between FF-MIP and FF-NIP was plotted in Figure 5d, revealing that the maximum value across all concentrations was reached at 12 min, followed by a sharp decline due to fiber saturation.

As Equation (1) describes, the output light beam depends on fiber length; at greater lengths, the output intensity decreases, but it is worth saying that with smaller lengths, less TAR can be adsorbed. With the purpose of studying the effect of fiber length, various immersions of the fiber in TAR solutions were carried out, covering segments of 28, 22, 18, and 12 mm of its length for 12 min of contact time.

**Figure 6:**
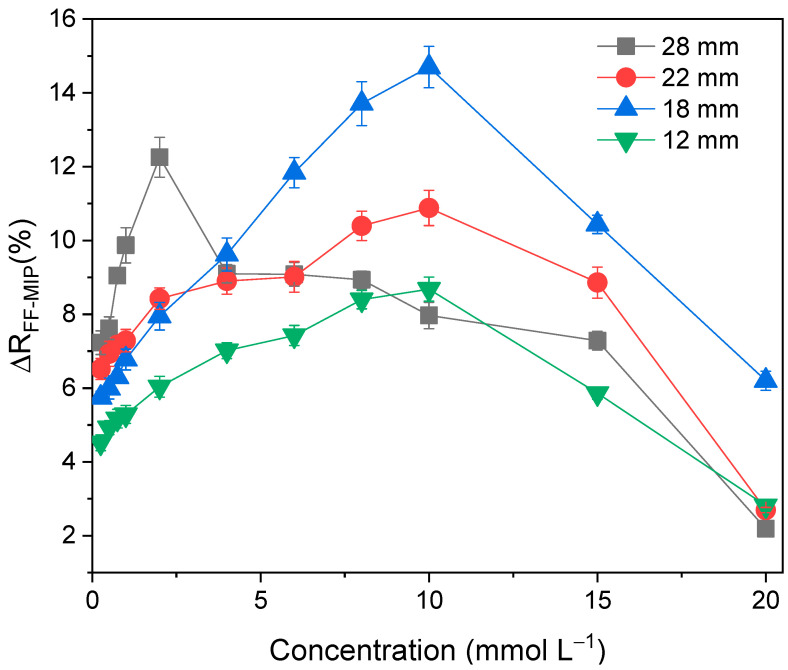
Reflectance variation profile of FF−MIP against tartrazine concentrations (0.25−20 mmol L^−1^) at different immersions of FF−MIP (12, 18, 22, and 28 mm) for 12 min. (n = 3).

**Figure 7:**
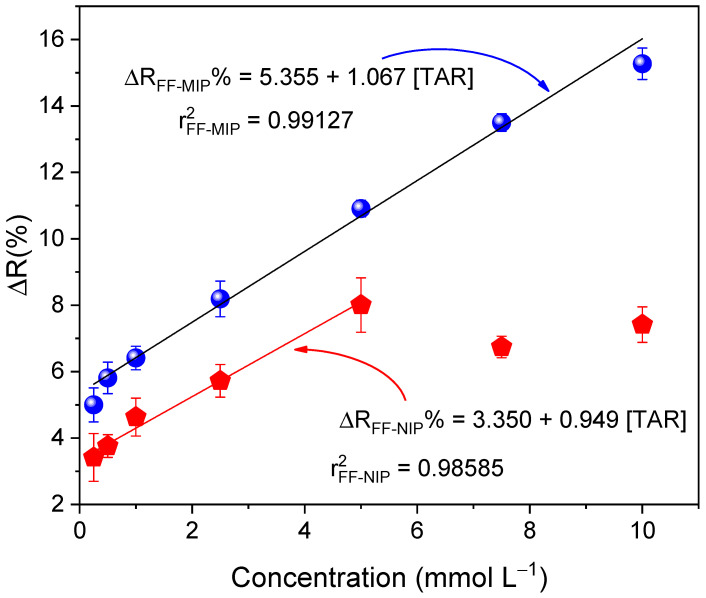
Reflectance variation profile of FF−MIP and FF−NIP at TAR concentrations (0.25−20 mmol L^−1^) at 22 mm immersions for 12 min of contact time. (n = 3).

Figure 6 was constructed from the results obtained from the described assay. For FF-MIP total immersion (28 mm), at concentrations higher than 2 mmol L^−1^, the reflectance variation decrease became evident as the fiber was reaching saturation, as described previously. In contrast, for partial immersions (12, 18, and 22 mm), the effect occurred at concentrations greater than 10 mmol L^−1^. As the refractive index of the fiber (*n_f_*) increases due to the greater amount of adsorbed tartrazine, the penetration distance (*d_p_*) decreases, as does the extinction coefficient of the evanescent wave, *ξ(n_s_)*, resulting in higher light intensity returning to the spectrophotometer, and consequently, lower reflectance variation. What is more, the working range was expected to amplify as the fiber length decreased; however, it is crucial to consider that, by decreasing the fiber length, the amount of analyte that it can adsorb is also reduced, leading to a decrease in the observed signal. The immersion length of 22 mm was selected as the optimal parameter for reflectance measurements in subsequent studies, as it demonstrated higher sensitivity and a wider working range. Finally, the reflectance variation (Δ*R%*) of FF-MIP and FF-NIP at different concentrations (Figure 7) showed that FF-NIP presented a rapid saturation as the reflectance variation reached its maximum at 5 mmol L^−1^, then the Δ*R%* remained constant until 20 mmol L^−1^, possibly due to the agglomeration of the polymer on the fiber, the adsorbed TAR had minimal influence on FF-NIP refractive index, suggesting that not all adsorption sites were occupied. Upon reaching saturation, the refractive index of FF-NIP surpassed the solvent refractive index, resulting in a decrease in Δ*R%*.

### 3.3. Reusability of the Fiber Sensor and Repeatability of the Obtained Signal

**Figure 8:**
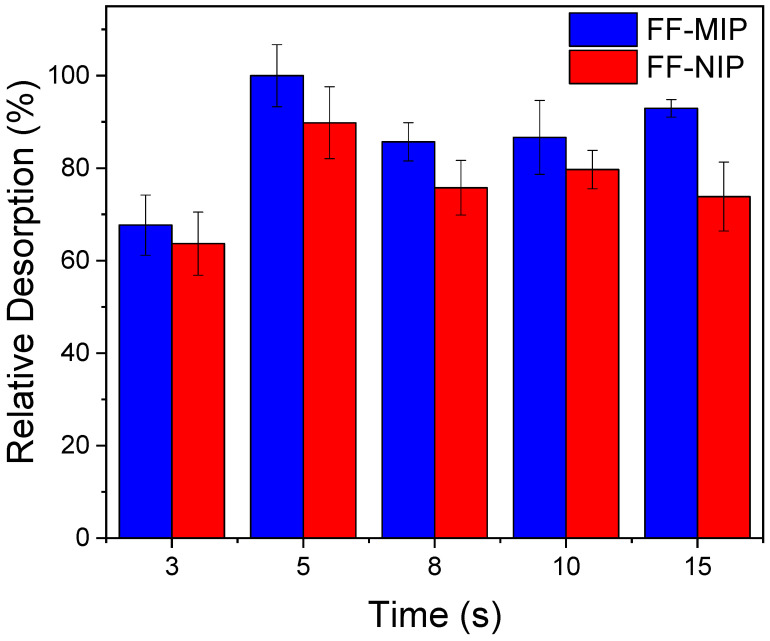
Relative desorption of TAR at different times after 12 min of contact time on the adsorption of 1.0 mmol L^−1^ of tartrazine (n = 3) employing FF-MIP and FF-NIP fibers with 22 mm length immersion.

To evaluate fiber reusability, a desorption test was carried out. Figure 8 illustrates that maximum desorption was achieved within just 5 s; at longer intervals, the system reached the adsorption/desorption equilibrium. Additionally, no significant variations in the blank measurements via the reflectance of FF-MIP and FF-NIP (<2%) were observed before and after the desorption test. This observation confirms the swift removal of the analyte, validating the existence of rapid mass transfer. Highlighting the prompt and complete removal of the analyte facilitates the immediate reusability of the sensor for successive analyses using a single fiber.

To verify the reusability aspects of the fiber, a repeatability assessment was conducted. The results are detailed in Table 1, indicating that the *RSD_Horwitz_* (1.539–2.130%) for each TAR concentration exceeded the RSDs of the FF-MIP fiber (1.122–1.784%). The obtained variability was within acceptable limits (*RSD_exp_* < *RSD_Horwitz_*), indicating satisfactory repeatability in terms of FF-MIP. However, for FF-NIP, the measurement repeatability was rejected as *RSD_exp_* exceeded *RSD_Horwitz_*.

### 3.4. Analysis of Interference and Selectivity

**Figure 9:**
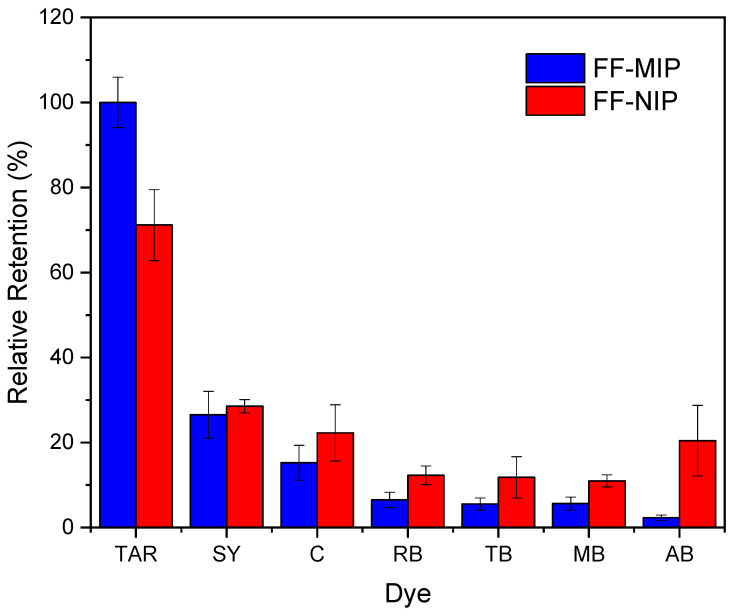
Analysis of selectivity toward TAR recorded as the relative retention (%) for each dye evaluated based on the application of the FF-MIP and FF-NIP (n = 3).

The interference analysis was performed using some dyes that have the ability to cause potential interference in TAR detection. The plot of the relative retention for each dye is shown in Figure 9. These results were satisfactory since there was a greater signal of TAR compared to the other dyes. Furthermore, for the analysis of the selectivity of the FF-MIP, the molecular imprinting factor (α) and the selectivity factor (β) were determined; the results obtained were found to be satisfactory. As can be observed in Table 2, the imprinting factor (α) obtained for the analyte of interest (TAR) was above that of the potential interferents; in addition, a higher selectivity factor (β) was obtained for TAR, with β values ranging from 1.501 to 12.545, thus confirming the high selectivity of the FF-MIP material. The recognition mechanism of FF-MIP cavities could be attributed to electrostatic interactions between the positively charged nitrogen (N+) of the functional monomer AA and the negatively charged oxygen (O−) of TAR, as depicted in Figure 1. This interaction appeared to be more pronounced in comparison to the studied interfering dyes, suggesting a stronger affinity. Additionally, repulsive interactions could occur between AA and the interfering dyes, such as RB and TB, both of which possess N+, and no cavities are filled, leading to no recognition or discrimination of these dyes. Based on the obtained results, the pronounced selectivity of the proposed material becomes apparent, primarily stemming from the distinct characteristic of the MIP employed as the recognized sensing phase.

Table 3 presents a comparative analysis of some relevant experimental data obtained from the application of the FF-MIP/UV-Vis spectrophotometry reflectance method proposed in this study and those recorded using other methods reported in previous studies on optical sensors for tartrazine quantification [23,46,47]. As can be noted, the results obtained, in terms of efficiency and selectivity, from the application of our proposed method were quite similar and clearly comparable to those recorded for other methods employed in previous studies, even though the imprinting factor presented by Bereli [47] was the highest due to the employed method, the selectivity factors are comparable; essentially, this shows that our proposed FF-MIP/UV-Vis spectrophotometry reflectance method has great potential for application in TAR quantification.

### 3.5. Response Profile of the FF-MIP and the Application of the Sensor in Real Samples

**Figure 10:**
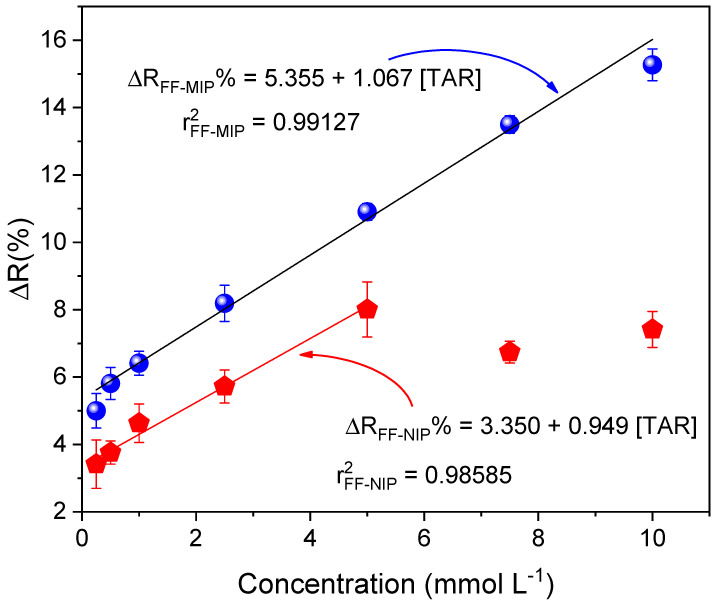
Analytical curves obtained for the FF−MIP and FF−NIP based on the application of TAR concentration range of 0.2 to 8 mmol L^−1^ (n = 6).

Analytical calibration curves of the FF-MIP and FF-NIP were plotted under the following conditions: a contact time of 12 min and an immersion length of 22 mm at TAR concentrations ranging from 0.25 to 10 mmol L^−1^; the curves are given in Figure 10. As can be observed, FF-MIP exhibited higher sensitivity to tartrazine as the slope was higher than that of FF-NIP. The proposed FF-MIP detection method recorded the following linear equation: ΔR_FF-MIP_% = 5.335 + 1.067 [TAR], r^2^ = 0.99127, n = 6, with a linear range of 0.25–10 mmol L^−1^, and LOD and LOQ values of 0.075 and 0.250 mmol L^−1^, respectively; for FF-NIP, the linear equation was as follows: ΔR_FF-MIP_% = 3.350 + 0.949 [TAR], r^2^ = 0.98585, n = 6, with a linear range of 0.25–5 mmol L^−1^, another difference between FF-MIP and FF-NIP responses was the linear range, where the effect of fiber saturation presented at lower concentrations, as discussed in the previous section. To test the applicability of the FF-MIP, five different water samples spiked with tartrazine were tested using the proposed UV-Vis spectrophotometry reflectance method and the HPLC reference method. Minimal sample preparation procedures were employed (adjusting pH to 3 with acid solution); the results obtained are shown in Table 4. No significant differences were observed between the two methods (*p* > 0.05 for *t*-test at 95% confidence level); hence, one can conclude that the proposed method is comparable to the HPLC method.

The findings of this study evidently point to the efficiency and suitability of the proposed FF-MIP sensor when applied for tartrazine quantification in water samples. The sensor requires minimal sample preparation and the use of less costly equipment.

## 4. Conclusions

A molecularly imprinted polymer (MIP) for the quantification of tartrazine was successfully synthesized on the surface of an optical fiber. The MIP was employed as a recognition material on the functionalized fiber (FF), and the FF-MIP material was used in conjunction with UV-Vis spectrophotometry to successfully quantify tartrazine (TAR) in real water samples. Under optimal conditions, the FF-MIP sensor exhibited good linearity for tartrazine determination in the range of 0.25 to 10.0 mmol L^−1^, with a determination coefficient (r^2^) of 0.9913. The limit of detection (LOD) and limit of quantification (LOQ) obtained were 0.075 and 0.250 mmol L^−1^, respectively. The FF-MIP material exhibited higher selectivity toward the analyte (TAR) compared to some interfering dyes. The use of functionalized optical fiber, combined with the high selectivity of MIP, provides a simple and portable material that is cost-effective and highly efficient for monitoring dyes in real water samples. Additionally, the material can be reused multiple times since the analyte can be easily removed. Thus, this feature endows the material with immediate usability post-synthesis. The proposed method has great potential to be used as a feasible alternative to time-consuming methods for the quantification of tartrazine in real water samples. 

## Figures and Tables

**Table 1:** Repeatability test of reflectance signal in FF-MIP and FF-NIP sensors. (n = 10).

Fiber (TAR Concentration)	$\bar{x}{\;}^{a}$	s ^b^	*RSD_exp_*	*RSD_Horwitz_*
FF-MIP (1.0 mmol L^−1^)	1.009	0.018	1.784	2.080
FF-NIP (1.0 mmol L^−1^)	0.861	0.057	6.620	2.130
FF-MIP (2.5 mmol L^−1^)	2.508	0.043	1.715	1.813
FF-NIP (2.5 mmol L^−1^)	2.609	0.159	6.092	1.803
FF-MIP (7.5 mmol L^−1^)	7.454	0.083	1.112	1.539

^a^ Average of 10 data points for the evaluated fiber. ^b^ Variance of 10 data points for the evaluated fiber.

**Table 2:** Parameters of selectivity toward TAR based on the application of the proposed FF-MIP sensor with some potential interfering dyes.

Dye	Structure	Selectivity Parameters	
		α (MIP/NIP)	β (Analyte/Interferent)
TAR	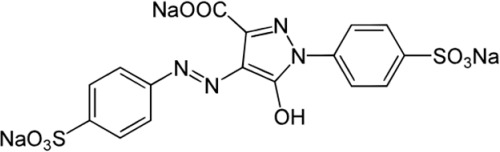	1.405	-
SY	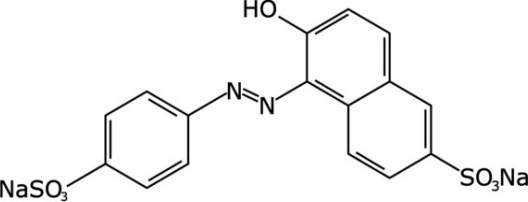	0.936	1.501
C	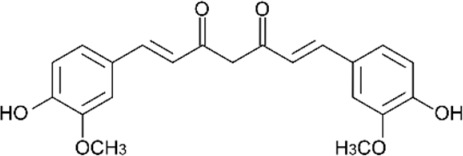	0.684	2.054
RB	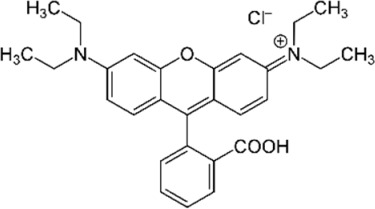	0.692	2.030
TB	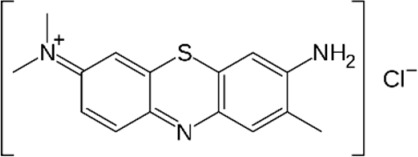	0.468	3.002
MB	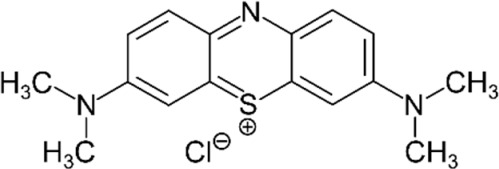	0.513	2.739
AB	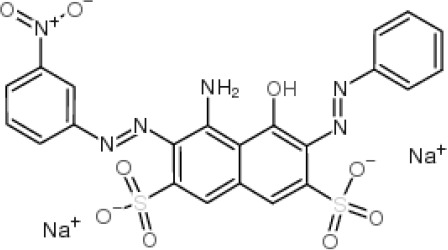	0.112	12.545

**Table 3:** Comparative analysis of our proposed method and other optical methods employed in previous studies reported for the quantification of TAR in the literature.

Method	Material	Imprinting Factor (α)	Selectivity (β)	Reference
Reflectance Spectrophotometry	Functionalized Silica Optical Fiber	1.41	1.501–12.545	Present study
Reflectance Spectrophotometry	SiO_2_@MIP	1.27	1.4–264	Ruiz, 2021 [23]
UV-Vis Spectrophotometry	Fe_3_O_4_@SiO_2_	2.46	2.035–2.253	Foroughirad, 2018 [46]
Surface Plasmon Resonance	PHEMAH * thin films	6.20	1.196–7.207	Bereli, 2021 [47]

* PHEMAH: Poly(hydroxyethyl methacrylate-N-methacryloyl-(L)-histidine methyl ester).

**Table 4:** Analysis of efficiency of our proposed method in comparison with the HPLC method (reference) using 5 real water samples: two different samples of drinkable water (A—pipe water; B—commercial bottled water), two different samples of river water (C—Cachi River; D—Millpu River) and one sample of wastewater (E); all the samples were obtained from the district of Huancaraya, Ayacucho, Peru (n = 3).

Sample (Symbol)	Method ^a^	
	HPLC ^b^	Proposed (FF-MIP) ^c,d^
Drinkable Water 1 (A)	1.141 ± 0.007	1.173 ± 0.054
Drinkable Water 2 (B)	1.281 ± 0.004	1.215 ± 0.083
Cachi River Water (C)	1.056 ± 0.003	1.059 ± 0.057
Millpu River Water (D)	1.071 ± 0.010	1.078 ± 0.067
Waste Water (E)	1.062 ± 0.003	1.073 ± 0.051

^a^ Concentrations in mmol L^−1^. ^b^ Values of TAR in five water samples obtained by the HPLC (reference method). ^c^ Values of TAR in five water samples obtained from the application of the proposed method. ^d^ Critical t-value = 4.303, calculated t_A_ = 1.018, t_B_ = 1.376, t_C_ = 0.091, t_D_ = 0.179 and t_E_ = 0.373, respectively to samples A, B, C, D, and E to *p* > 0.05 for *t*-test at 95% confidence level.

## Data Availability

The raw/processed data required to reproduce these findings are available upon request to the corresponding author. Our Institutions protect the data.

## Supplementary Materials

The following supporting information can be downloaded at: https://www.mdpi.com/article/10.3390/polym16060733/s1, Figure S1: Images of the (a) optical fiber, (b) functionalized optical fiber, and (c) functionalized optical fiber with MIP on the surface; Figure S2: FTIR spectra of MIP and NIP bulk; Figure S3: TGA and DTG thermographs obtained for the bulk (a) MIP and (b) NIP materials.
