# Supplementary material for: Development of an Optical Sensor Using a Molecularly Imprinted Polymer as a Selective Extracting Agent for the Direct Quantification of Tartrazine in Real Water Samples
Source: Polymers (Basel). 2024 Mar 7;16(6):733. doi: 10.3390/polym16060733 (PMC10975386; doi:10.3390/polym16060733)
Supplement: Supplementary file 1 [file polymers-16-00733-s001.zip › polymers-2895670-supplementary.pdf]

Supplementary Content

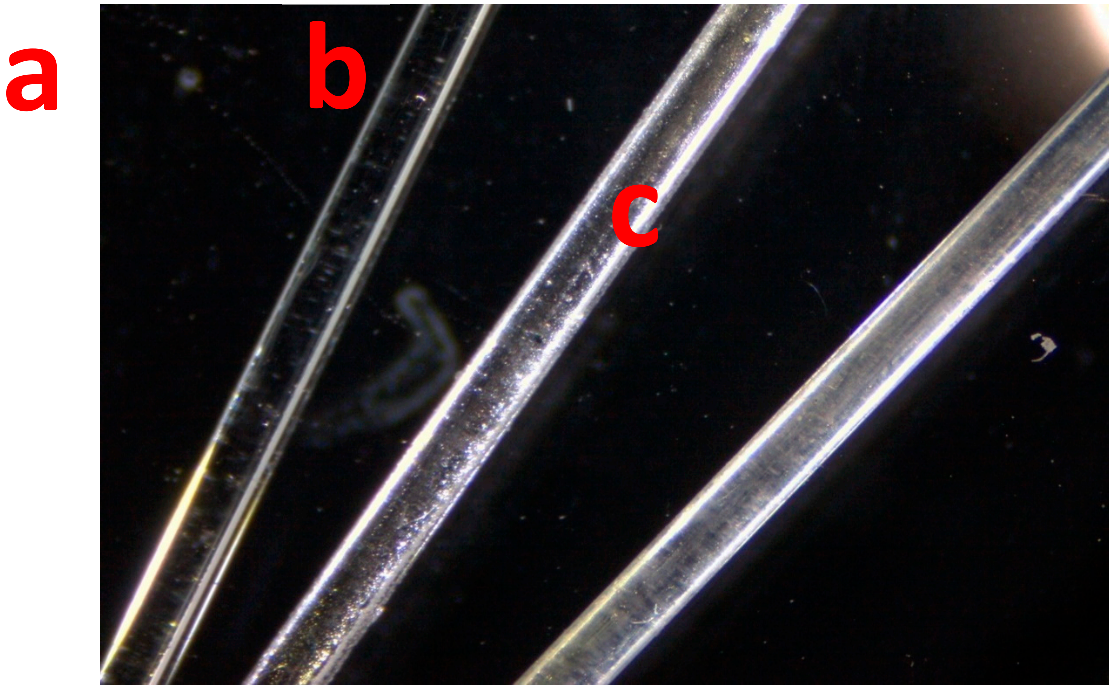

Figure S1. Images of the (a) optical fiber, (b) functionalized optical fiber, and (c) functionalized optical fiber with MIP on the surface.

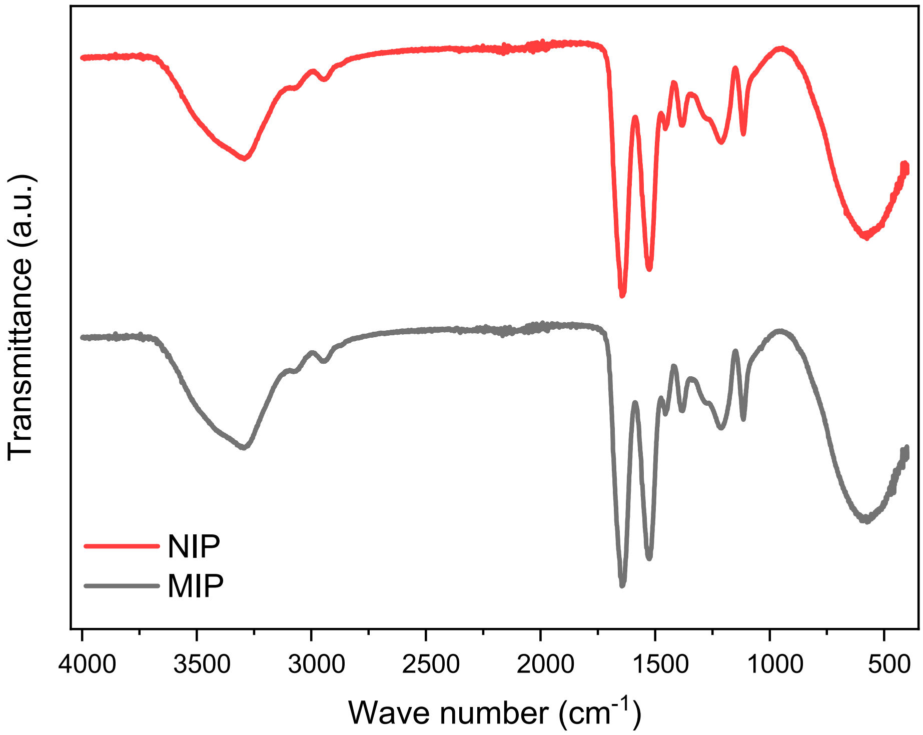

Figure S2. FTIR spectra of MIP and NIP bulk.

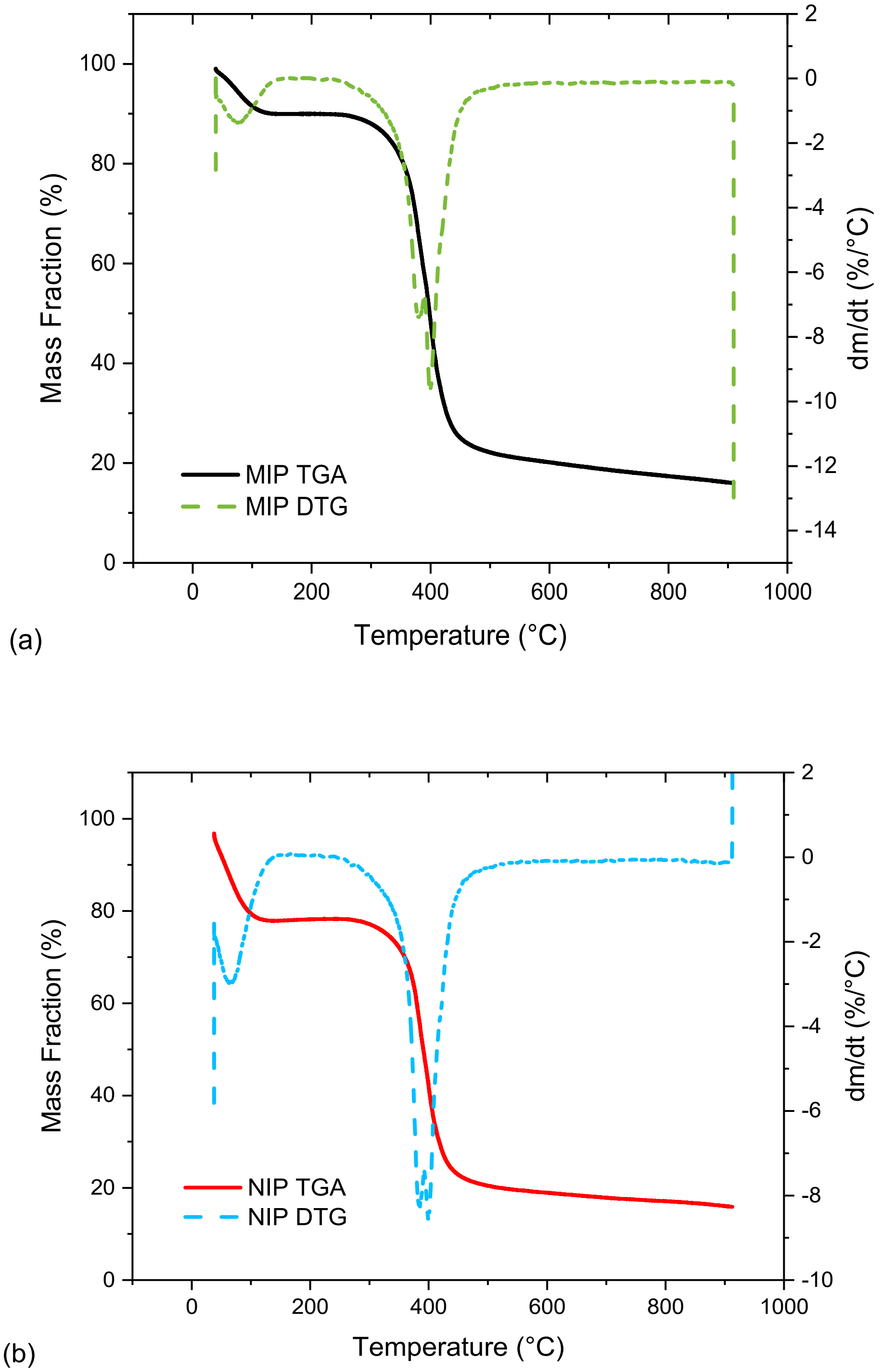

Figure S3. TGA and DTG thermographs obtained for the bulk (a) MIP and (b) NIP materials.
